# Procedural performance between two cryoballoon systems for ablation of atrial fibrillation depends on pulmonary vein anatomy

**DOI:** 10.1002/joa3.12842

**Published:** 2023-03-17

**Authors:** Vincent Menger, Michael Frick, Ahmad Sharif‐Yakan, Mahdi Emrani, Matthias Daniel Zink, Andreas Napp, Nikolaus Marx, Michael Gramlich

**Affiliations:** ^1^ Department of Cardiology University Hospital RWTH Aachen Aachen Germany

**Keywords:** atrial fibrillation, cryoablation, pulmonary vein anatomy

## Abstract

**Background:**

Cryoballoon ablation is a first‐line therapy for atrial fibrillation. We compared the efficacy and safety of two ablation systems and addressed the influence of pulmonary vein (PV) anatomy on performance and outcome.

**Methods:**

We consecutively enrolled 122 patients who were planned for first‐time cryoballoon ablation. Patients were assigned 1:1 for ablation with the POLARx or the Arctic Front Advance Pro (AFAP) system and followed‐up for 12 months. Procedural parameters were recorded during the ablation. Before the procedure, a magnetic resonance angiography (MRA) of the PVs was generated and diameter, area, and shape of each PV ostium were assessed. We applied an evaluated PV anatomical scoring system on our MRA measurement data ranging from 0 (best anatomical combination) to 5.

**Results:**

Procedures performed with POLARx were associated with shorter time to balloon temperature −30°C (*p* < .001), lower balloon nadir temperature (*p* < .001), and longer thawing time till 0°C (*p* < .001) in all PVs, however, time to isolation was similar. We observed a decreasing performance with each increase in the score for the AFAP, whereas the POLARx performed constant regardless of the score. At 1 year, AF recurred in 14 of 44 patients treated with AFAP (31.8%) and in 10 of 45 patients treated with POLARx (22.2%) (hazard ratio, 0.61; 95% CI 0.28 to 1.37; *p* = .225). There was no significant correlation between PV anatomy and clinical outcome.

**Conclusion:**

We found significant differences in cooling kinetics, especially when anatomical conditions are difficult. However, both systems have a comparable outcome and safety profile.

## 1 INTRODUCTION

Cryoballoon (CB) ablation for pulmonary vein isolation (PVI) as the first‐line therapy is superior to antiarrhythmic drugs for maintaining sinus rhythm in patients with symptomatic, paroxysmal atrial fibrillation (AF).^1^, ^2^ Moreover, CB ablation is a reasonable first‐line therapy in patients with persistent AF and has proven to be a safe and effective treatment option.^3^, ^4^ Recently, a new cryoablation system (POLARx™, Boston Scientific) has entered the market. Recent studies have associated POLARx with shorter time to balloon temperature − 30 °C (T‐30), lower balloon nadir temperatures (NT), and longer balloon thawing time till 0 °C (TT0); however, time to isolation (TTI) was similar to AFAP.^5^, ^6^ Furthermore, POLARx appeared to be safe, efficacious, and demonstrated a comparable 1‐year outcome.^7^, ^8^, ^9^, ^10^ Biophysical parameters such as balloon NT and TT0 have been associated with durability of PVI after CB ablation.^11^, ^12^, ^13^ The aim of the present study was to compare the biophysical parameters as well as complications and outcome to the established Arctic Front Advance Pro™ (AFAP, Medtronic) ablation system. Pulmonary vein (PV) anatomy can affect cooling kinetics and CB ablation outcome.^14^, ^15^, ^16^, ^17^ A recent study demonstrated an association between cross‐sectional orifice area of the superior PVs and outcome after CB ablation in patients with paroxysmal AF.^9^ Normal PV anatomy was associated with a significantly improved outcome for both catheter systems.^9^ We addressed the influence of various PV anatomical characteristics on the cooling kinetics of both cryoablation systems by applying an established PV anatomy scoring system to preprocedural magnetic resonance (MR) angiography data.^14^

## 2 METHODS

This study was conducted in accordance with the Declaration of Helsinki and was approved by the local ethics committee. The authors had full access to and take responsibility for the integrity of the data and have read and agree with the manuscript as written.

### 2.1 Study design

This was a non‐randomized prospective single‐center study. We enrolled 122 consecutive patients who underwent a CB ablation for symptomatic AF in our center from November 2020 to May 2021. For the first 61 patients, the AFAP CB (Medtronic) was used. The following 61 procedures were performed by using the POLARx cryoablation system (Boston Scientific). The day before the ablation, a MR angiography of the pulmonary veins was generated and PV anatomy was evaluated. Procedural parameters (balloon NT, total freeze duration, T‐30, TTI, TT0, body time and fluoroscopy time), as well as acute safety and efficacy outcomes were recorded during the ablation.

### 2.2 Patient enrollment criteria

Briefly, we enrolled adults (>18 years of age) who underwent a first‐ever ablation for symptomatic paroxysmal or persistent AF. All patients provided written informed consent.

### 2.3 Cryoablation systems

The POLARx (Boston Scientific, Marlborough, MA, USA) is an upcoming cryoablation system, which largely resembles the well‐established AFAP (Medtronic, Minneapolis, Minnesota). Both systems consist of a CB catheter (POLARx™ / AFAP™), a steerable sheath (POLARSHEATH™ / FlexCath Advance™), a mapping catheter (POLARMAP™ / Achieve Advance™) and a console (SMARTFREEZE™ / CryoConsole™). Differences to the AFAP are mainly found in the steerable sheath, which offers a slightly greater angle of maximal deflection (155° in POLARSHEATH™ vs. 135° in FlexCath Advance™).^18^ The POLARx was designed to maintain stable pressure during all ablation phases aiming to prevent dislodgement of the CB.^18^ Furthermore, the SMARTFREEZE console offers the possibility to directly display esophageal temperature and diaphragm movement monitoring.

### 2.4 Cryoballoon ablation

Cryoablation was performed as described earlier.^19^ Briefly, the ablation was performed under deep sedation and right‐sided PVs were treated under phrenic nerve pacing. Introducing the 28‐mm CB via a steerable sheath (FlexCath Advance™, Medtronic/POLARSHEATH™, Boston Scientific), the mapping catheter (Achieve Advance™, Medtronic/PolarMap™, Boston Scientific) was used as a guidewire to position the CB proximal to the PV ostium. After inflation of the CB, occlusion was verified for each PV by injecting contrast medium through the inner lumen of the CB. We assessed TTI rigorously by two experienced operators during the ablations. The procedural end point was defined as persistent isolation (entry and exit block) of all PVs verified by the mapping catheter.^19^ For safety reasons, a maximum of three CB applications per PV were performed. The standard freeze duration was 180 s. In case of late TTI or unsatisfactory temperatures, the examiners were allowed to freeze up to 240 s. If PV isolation was not possible but the patients were in sinus rhythm in the follow‐up examination, no further interventions were performed. If patients were diagnosed with symptomatic recurrence of AF after a blanking period of 90 days , a second ablation was scheduled using a 3D mapping system and RF energy. At this point, however, the patients were no longer part of the study, as recurrence of AF was considered as therapy failure.

### 2.5 Thermal imaging

**FIGURE 1:**
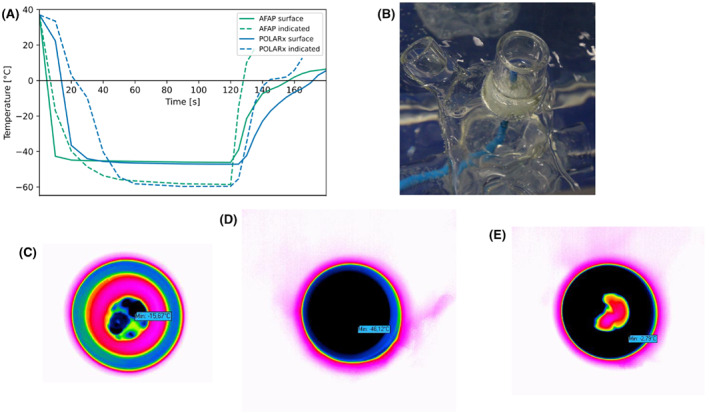
In vitro measurement of balloon surface temperature. (A) Balloon surface temperature of POLARx and AFAP vs. indicated console temperatures. Freeze duration was 120 s. (B), Picture of experimental set up. (C–E), Thermal images of cryoballoon surface while initiating (C), freezing (D), and thawing (E). AFAP, Arctic Front Advance Pro.

Aiming to prove correctness of the indicated balloon NTs, we measured the balloon surface temperature by thermal imaging. The experiment set up is shown in Figure 1. Briefly, in vitro freezes were performed with both CBs on a glass model of the left atrium and PVs in a tank filled with 10 liters of isotonic saline solution. Using a thermostat pump, we ensured a constant temperature of 37°C before every freeze. Balloon surface temperatures of the distal hemisphere of both CBs were measured every second by a thermal camera (Variocam high‐resolution research 685 s, Infratec, Germany, Hachenburg). Thermal images were retrospectively analyzed, and balloon surface nadir temperatures were detected with IRBIS® 3 professional as demonstrated in Figure 1.

### 2.6 MR imaging

MR imaging was performed 1 day before cryoballoon ablation on a 1.5 Tesla MR‐scanner (Philips Achieva, Philips Healthcare, Best, The Netherlands) with the patient in supine position. Depending on the patient's constitution, either a 5‐element cardiac synergy coil or a 32‐element cardiac coil was used for signal reception. After survey scanning, a standard contrast enhanced 3D‐angiography (T1‐weighted gradient echo sequence, parallel imaging with a sense factor of 3, TR: 2.3 ms, TE: 0.9 ms, flip angle: 30°, FOV of 500 × 500 × 150 mm, acquired voxel size of 1.5 × 1.5 × 2.4 mm, reconstructed to 1.0 × 1.0 × 1.2 mm) with bolus tracking to visualize the pulmonary veins and breath hold for respiratory compensation was performed. Contrast media was given intravenously (10 mL of Gadobutrol, Gadovist®, Bayer Vital GmbH, Germany), followed by a saline flush of 20 mL, both at 3 mL/s. Images were stored in DICOM format to the local institutions PACS.

### 2.7 MR image analysis

**FIGURE 2:**
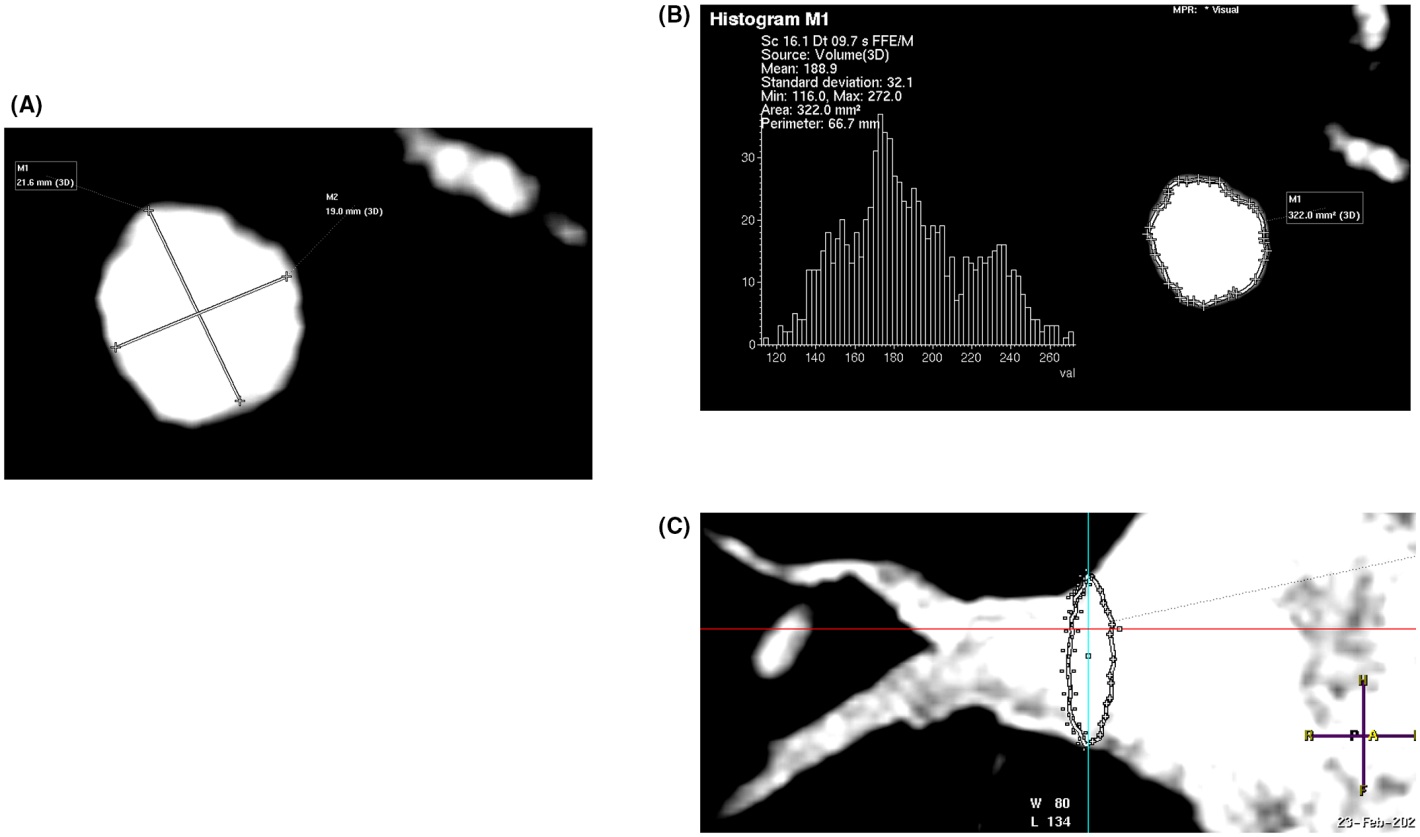
Magnetic resonance angiography of pulmonary veins. (A, B) Examples of the adapted perpendicular plane used for calculating ostial diameters (A) and area (B). (C) Evaluation of distance to branching.

All MR Images were first viewed in the axial, coronal, and transversal plane. Then, a multiplanar reconstruction software (Philipps IntelliSpace PACS, Philips Healthcare, Best, The Netherlands) was used to obtain the oblique view of each PV ostium. PV ostium was defined as the point of maximal inflection between LA‐wall and PV. PV ostial area, maximal (PVd max), and minimal diameters (PVd min) were measured. The ovality index was calculated as the ratio between the maximal and minimal diameter. The distance to branching was measured in reconstructed 2D‐Images as shown in Figure 2. It was defined as the distance between PV ostium and the first branch leaving the vein and was expressed in millimeter. Using these parameters, an anatomical performance score was evaluated for each vein as described earlier.^14^ Briefly, the score was calculated by assigning one point for each of the following anatomical characteristics: PVd max ≤19.5 mm; PVd min ≤14.0; PV area ≤ 240 mm^2^; PV trunk length ≤ 24.0 mm; and PV ovality ≥1.45. A score of 0 is associated with perfect anatomical conditions for the 28‐mm CB, whereas a score of 5 represents difficult anatomical conditions for the 28‐mm CB. A NT cut‐off of −48°C was defined as satisfactory for creating durable lesions around the PV antrum.^20^

### 2.8 Follow‐up

After the procedure, patients were typically observed overnight and discharged the following day. All patients received 72‐h Holter monitoring at 3‐, 6‐, and 12‐ months post‐ablation. The first 90 days after procedure were considered as a blanking period for all subjects. The documented recurrence of AF lasting longer than 30 s was considered as therapy failure.

### 2.9 Statistical analysis

Continuous variables are expressed as mean ± SD and categorical variables are expressed as number and percentage. All continuous variables were tested for normal distribution by D'Agostino's K2 test. The χ^2^ test and Fisher's exact test were used for comparing categorical variables. Welch's t test was used for comparison of normally distributed continuous variables. The non‐parametric Mann–Whitney U‐test was used where data were not normally distributed. Separate univariable Cox proportional hazard models were used to identify the clinical and anatomical factors which were associated with the recurrence of AF. Unadjusted survival curves were estimated by Kaplan–Meier analysis and compared using the log‐rank test. All statistical tests were performed two‐sided. All *p*‐values less than .05 were considered as statistically significant. Statistical analyses were performed using GraphPad Prism 9.

## 3 RESULTS

### 3.1 Patients

Between November 2020 and May 2021, a total of 122 patients were enrolled in this study. All patients were undergoing their first PVI procedure. The first 61 patients received treatment with the AFAP CB. The following 61 procedures were performed using the POLARx cryoablation system. The AFAP group had a higher proportion of patients using ACE inhibitors and a higher proportion of patients suffering from diabetes mellitus compared with the POLARx group. Other baseline characteristics were balanced in the two groups (Table 1). The median age of all patients included was 64.1 years and 59% of all subjects were men. The average proportion of patients with persistent AF was 31.1%. At baseline, mean CHA_2−_DS_2_‐VASc‐Score was 2.4 and left ventricular ejection fraction was 53.4%. In both groups, 4 patients (6.6%) had a common ostium on the left side. Only 1 patient (1.6%) in the POLARx group had a common ostium of the right‐sided pulmonary veins. The baseline characteristics for both groups are listed in Table 1.

**TABLE 1:** Demographic and clinical characteristics of patients included in the study.

	AFAP™ (*n* = 61) mean ± SD/*n* (%)	POLARx™ (*n* = 61) mean ± SD/*n* (%)	*p*‐value
Clinical characteristics			
Age, y	64.8 ± 12.0	63.3 ± 11.8	.55
Female Sex	27 (44.3)	23 (37.7)	.46
Body mass index, kg/ m^2^	29.2 ± 6.3	29.5 ± 5.0	.49
Persistent AF	16 (26.2)	22 (36.1)	.24
EHRA‐Score	2.2 ± 0.6	2.2 ± 0.6	.65
CHA_2−_DS_2_‐VASc‐Score	2.5 ± 1.6	2.3 ± 1.6	.36
Echocardiographic parameters			
Left atrial diameter, cm	3.8 ± 0.6	3.9 ± 0.7	.77
Left atrial size, cm^2^	23.0 ± 5.2	24.3 ± 7.0	.97
LV ejection fraction, %	53.4 ± 5.0	53.3 ± 7.8	.35
LV hypertrophy	14 (23.0)	9 (14.8)	.25
Medical history			
Previous stroke	2 (3.3)	3 (4.9)	.65
Previous PCI	4 (6.6)	5 (8.2)	.73
Previous MI	3 (4.9)	2 (3.3)	.65
Chronic kidney disease	11 (18.0)	6 (9.8)	.19
Diabetes	13 (21.3)	4 (6.6)	**.02**
COPD	2 (3.3)	3 (4.9)	.65
OSAS	2 (3.3)	3 (4.9)	.65
Medication			
Class 1 antiarrhythmic drug	11 (18.0)	15 (24.6)	.38
Class 3 antiarrhythmic drug	9 (14.8)	5 (8.2)	.26
ACE inhibitor	25 (41.0)	12 (19.7)	**.01**
Beta blockers	48 (78.7)	52 (85.3)	.35
Anticoagulation	58 (95.1)	52 (85.3)	.07
PV anatomical features			
Left common ostium	4 (6.6)	4 (6.6)	1.00
Right common ostium	0 (0)	1 (1.6)	‐
Accessory PV right	9 (17.0)	11 (18.3)	.85

Abbreviations: ACE, angiotensin‐converting enzyme; AF, atrial fibrillation; COPD, chronic obstructive pulmonary disease; LV, left ventricular; MI, myocardial infarction; OSAS, obstructive sleep apnea syndrome; PV, pulmonary vein.

Bold value indicates *p* < 0.05 are statistically significant.

### 3.2 Procedural efficacy

A total of 477 PVs were targeted (AFAP: *n* = 239, POLARx: *n* = 238). In both groups, 98.7% of all pulmonary veins were isolated successfully (*p* = 1.00). TTI could be recorded in 161/239 (67.4%) of PVs in the AFAP group versus 181/238 (76.1%) of PVs in the POLARx group (*p* = .04). There were no significant differences for fluoroscopy time (AFAP: 12.1 ± 6.8 min, POLARx: 13.0 ± 7.7 min, *p* = .55) or balloon in body time (AFAP: 37.9 ± 9.2 min, POLARx: 37.8 ± 12.7, *p* = .28) (Table 2).

**TABLE 2:** Procedural parameters used for cryoballoon ablation.

Procedural parameters	Vein	AFAP	POLARx	*p*‐value
In body time, min	–	37.9 ± 9.2	37.8 ± 12.7	.28
Flouroscopy time, min	–	13.0 ± 7.7	12.1 ± 6.8	.55
Total freeze duration, s	LSPV	274.1 ± 99.1	289.9 ± 98.3	.34
	LIPV	226.3 ± 85.3	247.2 ± 107.2	.34
	RSPV	285.8 ± 64.8	218.1 ± 105.0	.09
	RIPV	188.2 ± 69.3	206.6 ± 67.6	.38
Nadir temperature	LSPV	−52.6 ± 4.8	−61.8 ± 5.9	**<.001**
	LIPV	−48.8 ± 6.0	−56.7 ± 6.8	**<.001**
	RSPV	−51.4 ± 6.3	−57.3 ± 7.2	**<.001**
	RIPV	−49.4 ± 6.2	−57.1 ± 7.5	**<.001**
Time to −30°C, s	LSPV	30.7 ± 6.8	26.9 ± 3.3	**<.001**
	LIPV	33.2 ± 9.9	27.7 ± 3.5	**<.001**
	RSPV	31.3 ± 9.6	28.4 ± 18.8	**<.001**
	RIPV	35.1 ± 10.7	27.2 ± 7.1	**<.001**
Time to isolation, s	LSPV	71.8 ± 66.1	70.2 ± 59.2	.78
	LIPV	86.7 ± 81.6	82.3 ± 79.6	.64
	RSPV	48.0 ± 37.4	92.6 ± 94.7	.09
	RIPV	68.4 ± 70.8	75.9 ± 61.9	.18
Time to 0°C, s	LSPV	12.6 ± 4.4	24.0 ± 6.9	**<.001**
	LIPV	10.2 ± 4.0	21.0 ± 7.0	**<.001**
	RSPV	12.2 ± 4.8	21.2 ± 6.2	**<.001**
	RIPV	10.4 ± 5.0	20.5 ± 5.8	**<.001**

Abbreviations: LIPV, left inferior pulmonary vein; LSPV, left superior pulmonary vein; RIPV, right inferior pulmonary vein; RSPV, right superior pulmonary vein.

Bold value indicates *p* < 0.01 are statistically significant.

### 3.3 Procedural performance parameters

We compared 61 procedures performed with the POLARx CB with the previous 61 consecutive PVI cases performed with the AFAP. There was no significant difference for total freeze duration in all veins (Table 2). Procedures performed with POLARx were associated with lower balloon NTs, shorter T‐30 and longer TT0; however, no differences for TTI could be observed (Table 2). Detailed information of procedural and biophysical parameters associated with cryoablation is presented in Table 2 for each PV.

### 3.4 In vitro performance parameters

Aiming to confirm correctness of indicated NTs, we performed in vitro measurements of surface temperatures of the distal hemisphere of both CBs. Most of our results on the bench match with our findings from procedure data. POLARx is associated with lower indicated balloon NT and longer TT0 (Table 3). However, we found differences for T‐30. In our in vitro experiment, the T‐30 was significantly shorter for AFAP (*p* < .001) (Table 3), which stands in contrast to our in vivo findings (Table 2). Balloon surface temperature measured by thermal imaging was significantly lower for POLARx (*p* < .001) (Table 3). Figure 1 demonstrates temperature profiles and thermal images of both CBs for a freeze duration of 120 s. Detailed information of all in vitro performance parameters is shown in Table 3. Based on our results, there is no indication for incorrect indicated balloon NTs.

**TABLE 3:** Results of in vitro measurement after 120 s freezing.

	AFAP (*n* = 10)	POLARx (*n* = 10)	*p*‐value
Indicated nadir temperature, °C	−58.8 ± 0.6	−61.4 ± 3.2	.012
Balloon surface temperature, °C	−46.2 ± 0.1	−47.2 ± 0.3	<.001
Time to −30°C, s	13.1 ± 1.0	37.3 ± 2.0	<.001
Time to 0°C, s	7.3 ± 0.5	20.5 ± 2.5	<.001

### 3.5 MR imaging

A total of 93 PVI procedures (AFAP: *n* = 44, POLARx: *n* = 49) with complete MR scan data were analyzed. Using multiplanar reconstruction software, ostial area, maximal and minimal diameter, ovality index and distance to first branching were calculated for each PV. Detailed information of PV anatomical features is presented in Table S1. In the AFAP group, ostial area as well as maximal and minimal diameter were significantly smaller for RIPV (Table S1). Additionally, PV ovality index was slightly higher for the RSPV in the AFAP group. Other pulmonary vein anatomical conditions were balanced for both groups (Table S1).

### 3.6 Anatomical performance score

**FIGURE 3:**
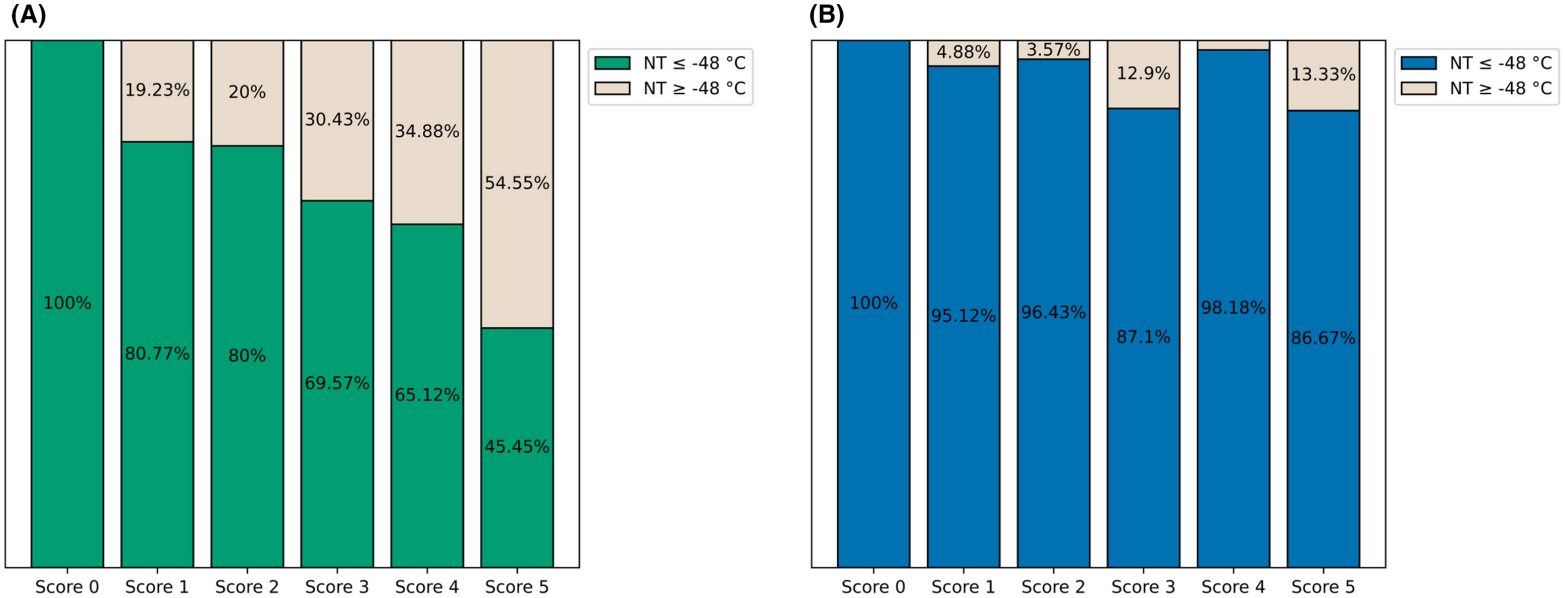
Percentage of satisfactory CB applications. This bar graph represents the percentage of satisfactory cryoballoon applications in every scoring group (defined as NT ≤−48°C). (A) shows results for AFAP, (B) for POLARx. AFAP, Arctic Front Advance Pro; NT, nadir temperature.

**FIGURE 4:**
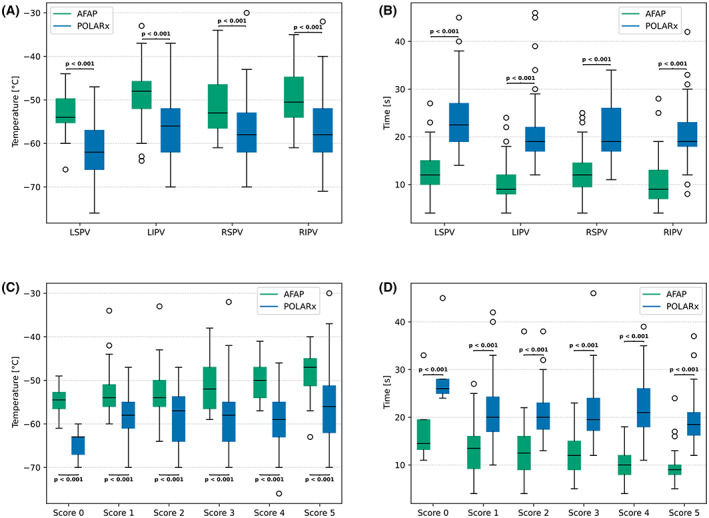
Procedural parameters and anatomical score performance. This boxplot shows balloon nadir temperatures (A) and balloon thawing time till 0°C (B) for each pulmonary vein. (C) shows balloon nadir temperatures and (D) balloon thawing time till 0°C for every scoring group. AFAP, Arctic Front Advance Pro; LIPV, left inferior pulmonary vein; LSPV, left superior pulmonary vein; RIPV, right inferior pulmonary vein; RSPV, right superior pulmonary vein.

Based on our results presented in Table S1, we calculated anatomical performance score as described by Borio et al.^14^ A balloon NT of −48°C was defined as satisfactory.^20^ A total of 360 PV scans were analyzed (AFAP: *n* = 170, POLARx *n* = 190). Results of the score applied to both groups are shown in Figure 3. There were no significant differences between the groups for all scores ranging from 0 to 3 (Figure 3). However, the proportion of freezes reaching satisfactory temperatures was significantly higher for the POLARx in the groups with a score of 4 or 5. In particular, in the group with a score of 4, AFAP could only reach satisfactory temperatures in 65% of all PVs, whereas POLARx reached −48°C in 98% of all veins (*p* < .001) (Figure 3). In the group with a score of 5, AFAP failed in 55% of all PVs to reach −48°C, while POLARx failed only in 13% of the targeted PVs in reaching the required temperature (*p* = .001) (Figure 3). The data suggest that the POLARx is much less affected by difficult anatomic conditions. Figure 4 presents reached balloon NTs and TT0 for both groups depending on the anatomical score and for each PV.

### 3.7 Follow‐up

**FIGURE 5:**
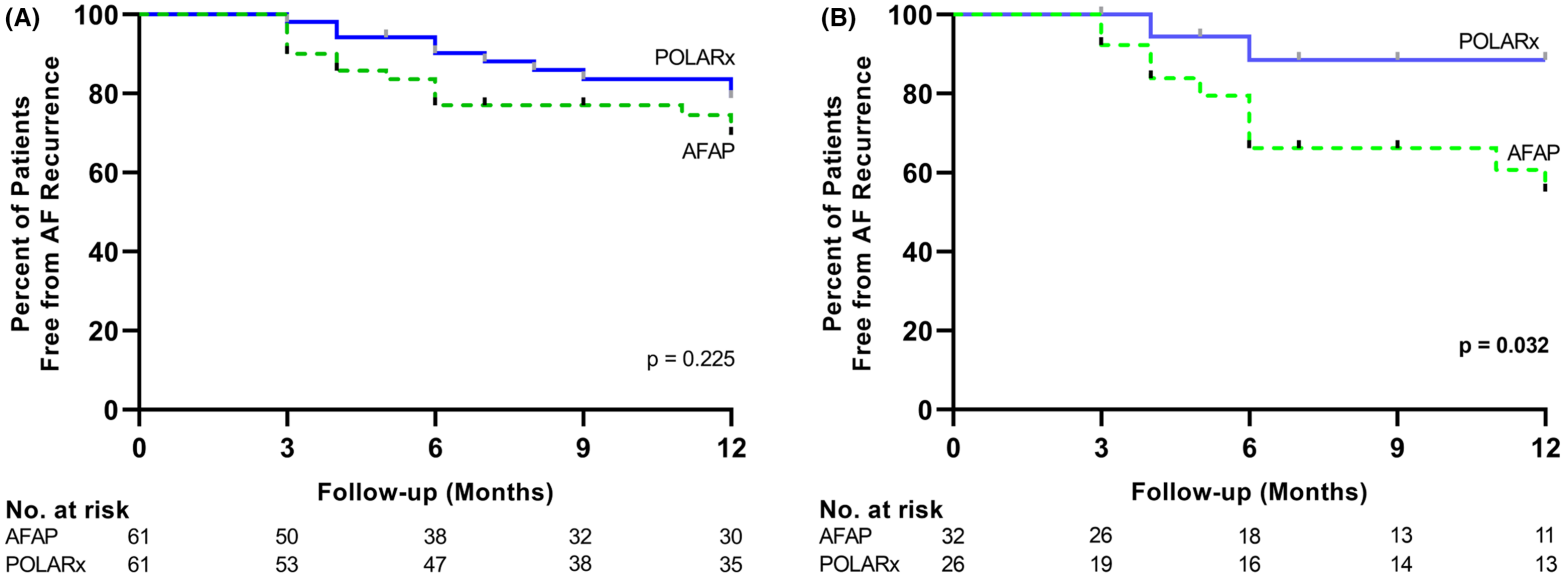
Freedom from Recurrence of Atrial Fibrillation over Time. Shown are Kaplan–Meier estimates for the overall study population (A) and the subgroup of patients with dilatated left atrium (la size>20 cm^2^) (B). The documented recurrence of atrial fibrillation lasting 30 seconds or longer between 91 and 365 days after cryoballoon ablation was considered as therapy failure. Tick marks indicate censored data. AFAP indicates Arctic Front Advance Pro.

A total of 89 patients attended 1‐year‐post‐ablation follow‐up (AFAP: *n* = 44, POLARx: *n* = 45). At least one documented AF episode lasting longer than 30 s had occurred in 14 of the 44 patients treated with the AFAP (31.8%) and in 10 of the 45 patients treated with POLARx (22.2%) (hazard ratio, 0.61; 95% confidence interval [CI] 0.28 to 1.37; *p* = .225) (Figure 5).

The results of the Cox‐hazard regression analysis are shown in Table S2. Left atrial size was associated with recurrence of AF for patients treated with AFAP (*p* = .005) (Table S2). In total, 36 patients with dilatated left atrium (Left atrial size >20 cm^2^) attended 1 year‐post‐ablation follow‐up (AFAP: *n* = 21, POLARx: *n* = 15). Atrial fibrillation was documented in 10 of the 21 patients treated with AFAP (47.6%) and in 2 of the 15 patients treated with POLARx (13.3%) (hazard ratio, 0.23, 95% CI, 0.07 to 0.71, *p* = .032) (Figure 5). There was no clear correlation between PV anatomy and clinical outcome for both systems (Table S2).

### 3.8 Safety

There was an overall low incidence of complications in both groups. Two transient phrenic nerve palsies were recognized in both groups, which recovered within the procedure (Table S3). One patient in the POLARx group suffered a persistent phrenic nerve palsy, which did recover before hospital discharge (Table S3). There were no cases of serious procedure‐related adverse events.

## 4 DISCUSSION

This clinical study compared safety, outcome, and acute procedural parameters of two competing cryoablation systems under consideration of PV anatomical conditions.

Main findings:

1. Both cryoablation systems appeared to be safe and effective.
2. Procedures performed with the POLARx were associated with shorter T‐30, longer TT0, and lower indicated NTs; however, TTI was similar.
3. Based on our in vitro measurements, there is no indication for incorrect indicated balloon NTs.
4. The POLARx appeared to be much less affected by difficult anatomical conditions than the AFAP.
5. There were no significant differences in clinical outcome between the treatment groups at 1‐year follow‐up.

With the market release of the POLARx CB, the question arises whether there is a superior cryoablation system. The POLARx largely resembles the incumbent AFAP, and workflow is mostly similar. Independently of the system used, procedural success was remarkably high with almost all veins isolated and a low rate of complications. In our study, we could not find significant differences for complications as well as for balloon in body time or fluoroscopy time, which leads to the conclusion that the safety profile is acceptable in both systems. Main differences were found in the gathered acute procedural parameters. We verified correctness of displayed procedural parameters for both cryoablation systems in an in vitro experiment. Some biophysical parameters have been associated with durability of PVI, such as balloon cooling rates, balloon NT, and balloon thawing times.^11^, ^12^, ^13^ From these parameters, balloon thawing time is believed to be the strongest predictor for durable PVI for the first‐ and second‐generation CB (Arctic Front, Arctic Front Advance, Medtronic).^11^, ^13^ In our study, the POLARx achieved significantly longer TT0 in all PVs. This might be a result of different material properties of both CBs. The material used for the POLARx is softer and believed to be more compliant which could lead to a better tissue‐balloon contact and occlusion of the PV antrum. But better sealing of the PV antrum cannot be the only reason for the elongated thawing phase. In our in vitro experiment, the POLARx was associated with significantly longer TT0 although both CBs sealed the PV model completely. The main reason for the significant differences in TT0 is that gas injection stops immediately after the end of freezing in the AFAP, whereas in the POLARx, a small amount of nitrous oxide is still circulating, which contributes greatly to the elongated thawing phase. Longer thawing phases are associated with additional cellular injury.^11^, ^21^ If this results in differences in outcome is currently unknown and needs further investigation. In our study, there were no significant differences between the groups in terms of clinical outcome. The POLARx demonstrated shorter T‐30 and lower NTs in all PVs in our study. However, TTI was comparable between both systems, which leads to the question if the indicated balloon NTs for the POLARx are incorrect. Technically, there is no difference in temperature measurement in both CBs. The temperature of refluent gas is measured at the proximal end of the CB and the location of thermocouples is similar. We also ensured correctness of indicated NTs by measuring the balloon surface temperature of the distal hemisphere of both CBs with a thermographic camera under in vitro conditions. Based on our measurements, there was no indication for incorrect indicated NTs. Given that POLARx has a faster cooling rate and reaches lower NTs, how can the missing difference in TTI be explained? First, the AFAP comes with 8 mm Tip length, whereas we exclusively used the POLARx with 5 mm tip length, resulting in a closer positioning of the mapping catheter to the PV ostium. Second, the signal quality of electrograms with the POLARMAP was felt to be excellent by us and other authors.^6^ Another relevant factor could be the loop size of the mapping catheter. The POLARMAP has a loop size of 20 mm, whereas the Achieve Advance mapping catheter we used has a loop size of 15 mm. This could improve the perception of signals from the POLARMAP, especially in large diameter PVs. The POLARMAP might reveal very small electrical connections, which might be missed by the Achieve Advance mapping catheter. One possible explanation is therefore that an earlier biological effect might be covered by better signal measuring, resulting in similar TTIs. The importance of this topic is demonstrated by the fact that several studies have shown that early TTI is the most powerful predictor of durable PVI.^11^, ^22^, ^23^ In both treatment groups, the TTI was longer than that in previous studies. Nevertheless, based on our long‐term outcome data, it can be seen that a good treatment success was achieved. Thus, we can only explain the discrepancy in TTI by the fact that we were very strict in our assessment of TTI. Balloon cooling rates and NTs were found to be weak predictors for durable PVI for the second‐generation CB (Arctic Front Advance, Medtronic) but a NT of −56°C was the best predictor for acute PVI for the POLARx in a recent study.^11^, ^13^, ^24^ As described previously, the data from our in vitro experiment widely match with our gathered procedural data. However, we found a significant difference for T‐30. Procedures performed with the POLARx were associated with significantly shorter T‐30. In our in vitro experiment, the AFAP demonstrated much faster cooling rates, although the POLARx reached overall lower NTs and balloon surface temperatures. This difference could be explained by the fact that the used glass model was designed for training of medical doctors with the second‐generation CB (Arctic Front Advance, Medtronic). Therefore, the metrics of the PVs in the model were most likely ideal for the AFAP. Moreover, the first 10 s of freezing do not take part in the cooling process of the POLARx according to Boston Scientific. In the early ablation phase, only a small amount of nitrous oxide is injected in the CB allowing the balloon to maintain stable pressure and preventing it to pop‐out of the PV. Another possible explanation is that there may have been less gas flow with the POLARx than in vivo owing to the specific experimental set up. The catheters were inserted into the water tank through a specially adapted cutout. To prevent the tank from losing water, we encased the catheters in a rubber seal. It could be possible that the POLARSHEATH was compressed by the rubber gasket because of its soft material properties and the gas flow was not as high as in vivo, resulting in a longer T‐30. In our study, the POLARx was much less affected by difficult anatomical conditions than the AFAP. We applied an evaluated PV anatomical scoring system on our MR angiography measurement data. In this scoring system, a score of 0 represents perfect anatomical conditions for the 28‐mm CB, whereas a score of 5 represents very difficult anatomical conditions for the 28‐mm CB. A NT cut‐off of −48°C was defined as satisfactory for creating durable lesions around the PV antrum for the second‐generation cryoballoon from Medtronic (Arctic Front Advance).^20^ At the time of our study, data for durable PVI with the POLARx system did not exist. However, we do not believe there is any reason why the temperature needs to be lower for the POLARx for durable lesions, as both systems display the temperature correctly, as demonstrated by our in vitro experiment. We could observe a decrease in the percentage of satisfactory CB applications for every increase in the score for the AFAP. The POLARx demonstrated independently of the score an overall high proportion of satisfactory CB applications. Our data suggest that there is no big difference in performance between these systems when anatomical conditions are easy for the 28‐mm CB, but POLARx might be superior when it comes to difficult anatomical conditions. Nevertheless, there was no significant difference in the overall long‐term outcome between the treatment groups. A significant difference in long‐term outcome was found only in patients with dilated left atrium (la size >20 cm^2^). In this subgroup, POLARx was associated with better long‐term treatment success. For reasons of the small number of subjects in this subgroup, further studies are needed to establish definitive results. Apart from atrial size, we could not identify other anatomical factors that influenced clinical outcome. It is probably difficult to find clear correlations between PV anatomy and clinical outcome, as the treatment success of CB ablation is also highly dependent on other factors such as the extent of atrial fibrosis.^19^ In addition, other factors could be relevant for ablation success, such as the localization of the transseptal puncture and the angle of the CB to the PV ostium. This will be object of future investigations.

### 4.1 Limitations

The validity of the study is mainly restricted by the study design as a nonrandomized single‐center study. We performed all procedures without using an esophageal temperature probe. Therefore, the effect of lower balloon NTs of the POLARx on esophageal temperature remains unclear.^5^ However, lower NTs in the POLARx group did not result in a higher rate of complications and no symptoms of esophageal irritation were described by subjects in the follow‐up examinations. Moreover, we applied an evaluated PV anatomical scoring system on our MR angiography measurement data but had slightly different measurement methods. While the cut‐off in the score was evaluated for PV trunk length, we measured distance to the first branch leaving the vein. Thus, the chosen cut‐off for PV trunk length of ≤24.0 mm could not be reached by a high proportion of all measured PVs. This resulted in a very small proportion of PVs reaching a score of 0. Lastly, we do not have information on the reconnection rate of the PVs. A large‐scale randomized clinical trial would be required to definitively show noninferiority or superiority between these technologies.

## 5 CONCLUSION

Both cryoablation systems are safe and effective, with similar long‐term outcomes. However, the POLARx system has faster cooling rates, lower balloon NTs and longer thawing times and is less affected by challenging anatomy. There is no correlation between PV anatomy and outcome for both systems. The choice of which system to use should be made according to the preferences of the treating physician.

## AUTHOR CONTRIBUTIONS

V.M. conceived and designed the analysis, planned, and carried out the in vitro experiment, collected the data, performed the statistical analysis, and wrote the paper. M.F. contributed data and analysis tools. A.S. contributed data. M.M. contributed data. M.Z. contributed data. A.N. contributed data. N.M. conceived and designed the analysis. M.G. conceived and designed the analysis, conceived, and planned the in vitro experiment, supervised the findings of this work, and corrected the manuscript.

## FUNDING INFORMATION

The work was supported by internal funds of the university RWTH Aachen.

## CONFLICT OF INTEREST STATEMENT

The authors have no relevant financial or nonfinancial interests to disclose.

## ETHICS APPROVAL STATEMENT

This study was conducted in accordance with the Declaration of Helsinki and was approved by the local ethics committee.

## PATIENT CONSENT STATEMENT

All patients provided written informed consent.

## DECLARATIONS

*Approval of the research protocol*: This study was conducted in accordance with the Declaration of Helsinki and was approved by the Ethics Committee of RWTH Aachen University (reference number: 075/21). *Informed Consent*: Informed consent was obtained from all individual participants included in the study. *Registry and the Registration No*.: The study was registered at the Center for Translational & Clinical Research Aachen (CTC‐A‐No.: 20–034). *Animal Studies*: N/A.

## Supporting information


Table S1.
Table S2.
Table S3.

## Data Availability

The authors had full access to and take responsibility for the integrity of the data and have read and agree with the manuscript as written. The data supporting the results of this study are available to reviewers upon request from the corresponding author. The data are not publicly available for privacy or ethical reasons.
