# Supplementary material for: Procedural performance between two cryoballoon systems for ablation of atrial fibrillation depends on pulmonary vein anatomy
Source: J Arrhythm. 2023 Mar 17;39(3):341–51. doi: 10.1002/joa3.12842 (PMC10264750; doi:10.1002/joa3.12842)

**Supplementary Material**

**Contents**

Supplementary Table S1

Supplementary Table S2

Supplementary Table S3

**TABLE S1** Anatomical characteristics of the pulmonary veins

|  | **LSPV** | | | **LIPV** | | | **RSPV** | | | **RIPV** | | |
| --- | --- | --- | --- | --- | --- | --- | --- | --- | --- | --- | --- | --- |
|  | **AFAP** | **PolarX** | **p** | **AFAP** | **PolarX** | **p** | **AFAP** | **PolarX** | **p** | **AFAP** | **PolarX** | **p** |
| Min PvD, mm | 14.0 ± 2.9 | 14.2 ± 3.2 | 0.83 | 11.5 ± 3.4 | 11.9 ± 3.2 | 0.44 | 14.1 ± 4.1 | 14.5 ± 3.4 | 0.43 | 14.2 ± 2.8 | 15.5 ± 2.9 | **0.02** |
| Max PvD, mm | 20.2 ± 3.2 | 21.3 ± 3.8 | 0.11 | 18.7 ± 3.8 | 17.9 ± 2.3 | 0.37 | 21.1 ± 3.6 | 20.3 ± 3.5 | 0.27 | 18.4 ± 2.9 | 20.2 ± 3.1 | **<0.01** |
| Ovality | 1.5 ± 0.3 | 1.5 ± 0.3 | 0.58 | 1.7 ± 0.4 | 1.6 ± 0.5 | 0.09 | 1.6 ± 0.3 | 1.4 ± 0.3 | **0.04** | 1.3 ± 0.2 | 1.3 ± 0.2 | 0.97 |
| Area, mm² | 232.5 ± 73.5 | 247.2 ± 89.9 | 0.65 | 183.3 ± 99.7 | 175.5 ± 56.6 | 0.62 | 251.7 ± 107.8 | 246.5 ± 82.6 | 0.84 | 216.9 ± 67.8 | 255.7 ± 77.6 | **0.01** |
| Branching, mm | 14.9 ± 8.1 | 17.4 ± 8.9 | 0.25 | 12.4 ± 6.6 | 11.3 ± 6.7 | 0.46 | 9.6 ± 5.7 | 9.6 ± 6.4 | 0.97 | 5.2 ± 3.6 | 6.5 ± 6.3 | 0.83 |

LSPV indicates left superior pulmonary vein; LIPV, left inferior pulmonary vein; RSPV, right superior pulmonary vein; RIPV, right inferior pulmonary vein; PvD, pulmonary vein diameter

PvD indicates pulmonary vein diameter

**TABLE S2** Results of regression analysis

|  | **Hazard Ratio** | | **95% CI** | | **p- value** | |
| --- | --- | --- | --- | --- | --- | --- |
|  | **AFAP** | **POLARx** | **AFAP** | **POLARx** | **AFAP** | **POLARx** |
| Female Sex | 0.9912 | 1.103 | 0.3261 - 2.852 | 0.2818 - 3.863 | 0.987 | 0.880 |
| Age (per year) | 0.9679 | 0.9821 | 0.9212 - 1.020 | 0.9400 - 1.036 | 0.205 | 0.457 |
| BMI (per unit) | 1.004 | 0.9932 | 0.9173 - 1.088 | 0.8703 - 1.123 | 0.933 | 0.915 |
| Persistent AF | 0.8467 | 0.7188 | 0.2828 - 3.089 | 0.2048 - 2.818 | 0.779 | 0.610 |
| EHRA (per point) | 1.103 | 0.5045 | 0.4462 - 2.758 | 0.1760 - 1.426 | 0.832 | 0.198 |
| CHA_2-_DS_2_-VASc-Score (per point) | 1.143 | 0.7999 | 0.7986 - 1.632 | 0.4972 - 1.232 | 0.459 | 0.327 |
| Echocardiographic parameters | | | | | | |
| Left atrial diameter, cm | 2.849 | 0.7740 | 1.093 - 7.188 | 0.2175 - 2.015 | **0.028** | 0.644 |
| Left atrial size, cm^2^ | 1.112 | 0.9975 | 1.024 - 1.194 | 0.8787 - 1.085 | **0.005** | 0.961 |
| LV ejection fraction, % | 1.082 | 1.012 | 0.9643 - 1.249 | 0.8713 - 1.177 | 0.258 | 0.878 |
| LV hypertrophy | 1.617 | 1.631 | 0.4403 - 10.39 | 0.2459 - 6.518 | 0.530 | 0.537 |
| PV anatomical features | | | | | | |
| Left common ostium | 1.673 | 1.376 | 0.07733 - 15.33 | 0.07457 - 7.343 | 0.674 | 0.762 |
| Accessory PV right | 3.710 | 1.600 | 0.7078 - 68.14 | 0.2379 - 6.645 | 0.212 | 0.559 |
| Score LSPV (per point) | 0.7218 | 1.185 | 0.4785 - 1.041 | 0.7429 - 2.002 | 0.093 | 0.492 |
| Score LIPV | 0.9289 | 1.551 | 0.5978 - 1.569 | 0.8489 - 3.740 | 0.758 | 0.225 |
| Score RSPV | 0.6484 | 1.469 | 0.3802 - 1.017 | 0.9004 - 2.661 | 0.075 | 0.154 |
| Score RIPV | 1.162 | 1.311 | 0.7165 - 2.032 | 0.7972 - 2.211 | 0.566 | 0.287 |
| Average Score | 0.6162 | 2.461 | 0.3543 - 1.086 | 1.078 - 6.505 | 0.088 | 0.052 |
| Maximum Score (all PVs) | 0.6890 | 1.633 | 0.4049 - 1.295 | 0.8436 - 4.658 | 0.198 | 0.238 |
| Periprocedural parameters | | | | | | |
| Balloon NT LSPV, °C | 0.9766 | 0.9415 | 0.8556 - 1.103 | 0.8412 - 1.052 | 0.711 | 0.289 |
| Balloon NT LIPV, °C | 0.9896 | 1.040 | 0.8989 - 1.090 | 0.9428 - 1.145 | 0.832 | 0.434 |
| Balloon NT RSPV, °C | 1.075 | 1.008 | 0.9848 - 1.166 | 0.9183 - 1.094 | 0.090 | 0.853 |
| Balloon NT RIPV, °C | 0.9449 | 1.036 | 0.8468 - 1.044 | 0.9483 - 1.116 | 0.283 | 0.399 |
| T-30 LSPV, s | 1.098 | 1.081 | 1.009 - 1.199 | 0.8944 - 1.306 | **0.021** | 0.412 |
| T-30 LIPV. s | 1.026 | 1.075 | 0.9744 - 1.064 | 0.8971 - 1.252 | 0.228 | 0.396 |
| T-30 RSPV, s | 1.040 | 0.9965 | 0.9923 - 1.079 | 0.8874 - 1.021 | 0.058 | 0.886 |
| T-30 RIPV, s | 1.029 | 1.009 | 0.9701 - 1.078 | 0.8878 - 1.067 | 0.285 | 0.827 |
| TTI LSPV, s | 1.008 | 1.004 | 0.9992 - 1.016 | 0.9895 - 1.015 | 0.050 | 0.535 |
| TTI LIPV, s | 1.004 | 1.005 | 0.9971 - 1.010 | 0.9974 - 1.011 | 0.198 | 0.138 |
| TTI RSPV, s | 1.000 | 0.9999 | 0.9778 - 1.015 | 0.9891 - 1.007 | 0.982 | 0.978 |
| TTI RIPV, s | 1.005 | 0.9966 | 0.9912 - 1.016 | 0.9788 - 1.008 | 0.403 | 0.640 |
| Time to 0 °C LSPV, s | 1.038 | 0.9907 | 0.9248 - 1.149 | 0.8735 - 1.097 | 0.503 | 0.870 |
| Time to 0 °C LIPV, s | 1.048 | 1.019 | 0.9042 - 1.185 | 0.9163 - 1.097 | 0.489 | 0.674 |
| Time to 0 °C RSPV, s | 0.8818 | 1.078 | 0.7499 - 1.022 | 0.9759 - 1.193 | 0.112 | 0.134 |
| Time to 0 °C RIPV, s | 1.078 | 1.092 | 0.9726 - 1.176 | 0.9580 - 1.247 | 0.113 | 0.186 |

**TABLE S3** Complications associated with cryoballoon ablation

| Complications | AFAP (n=61) | POLARx (n=61) | p |
| --- | --- | --- | --- |
| Transient phrenic nerve palsies | 2 (3.3) | 2 (3.3) | 1.00 |
| Persistent phrenic nerve palsies | 0 (0) | 1 (1.6) | 1.00 |
| Cardiac tamponade | 0(0) | 0(0) | - |
| Atrioesophageal fistula | 0(0) | 0(0) | - |
| Stroke | 0(0) | 0(0) | - |

**Distribution Plot TTI RSPV (Arctic Front Advance Pro)**


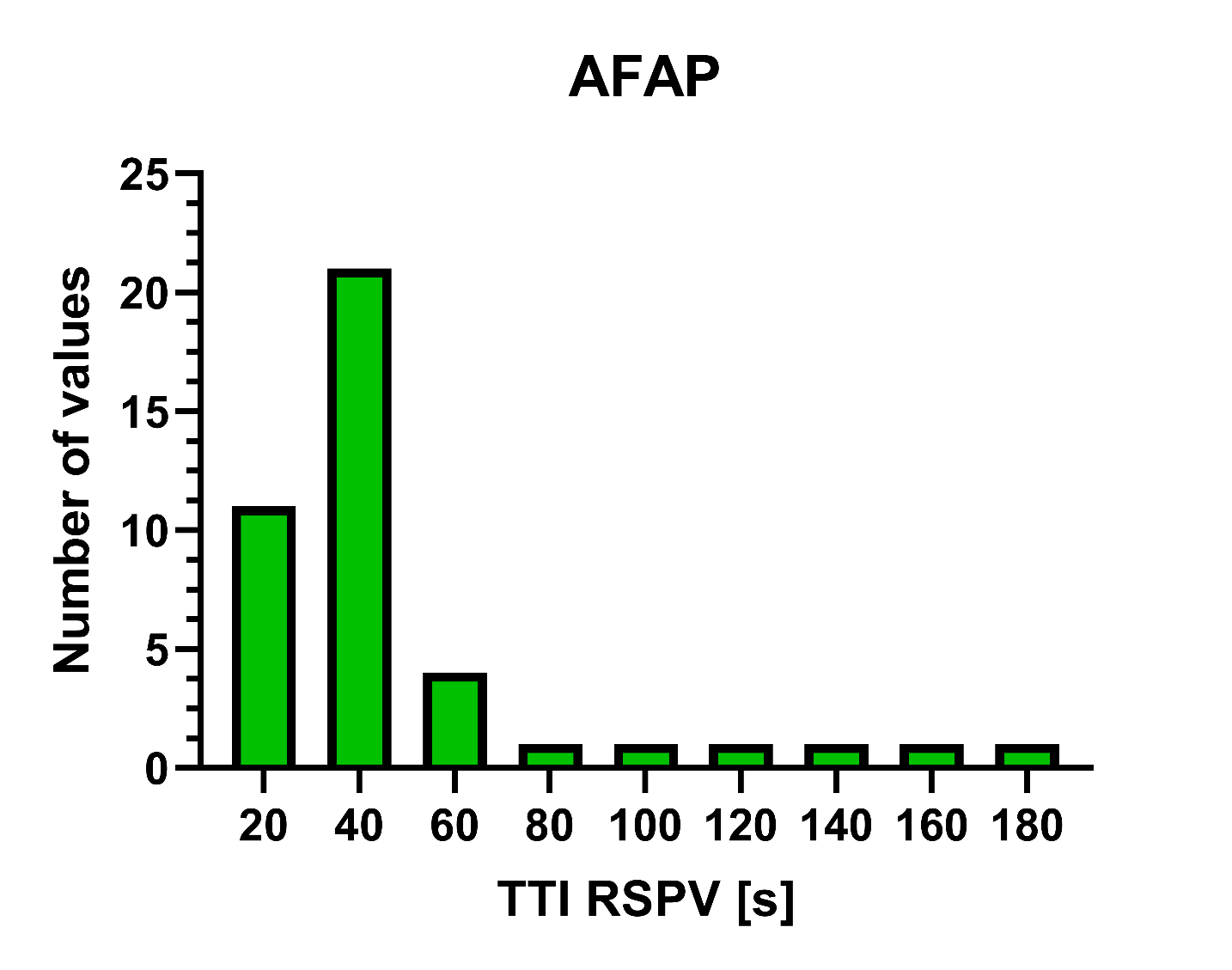


**Distribution Plot TTI RSPV (POLARx)**


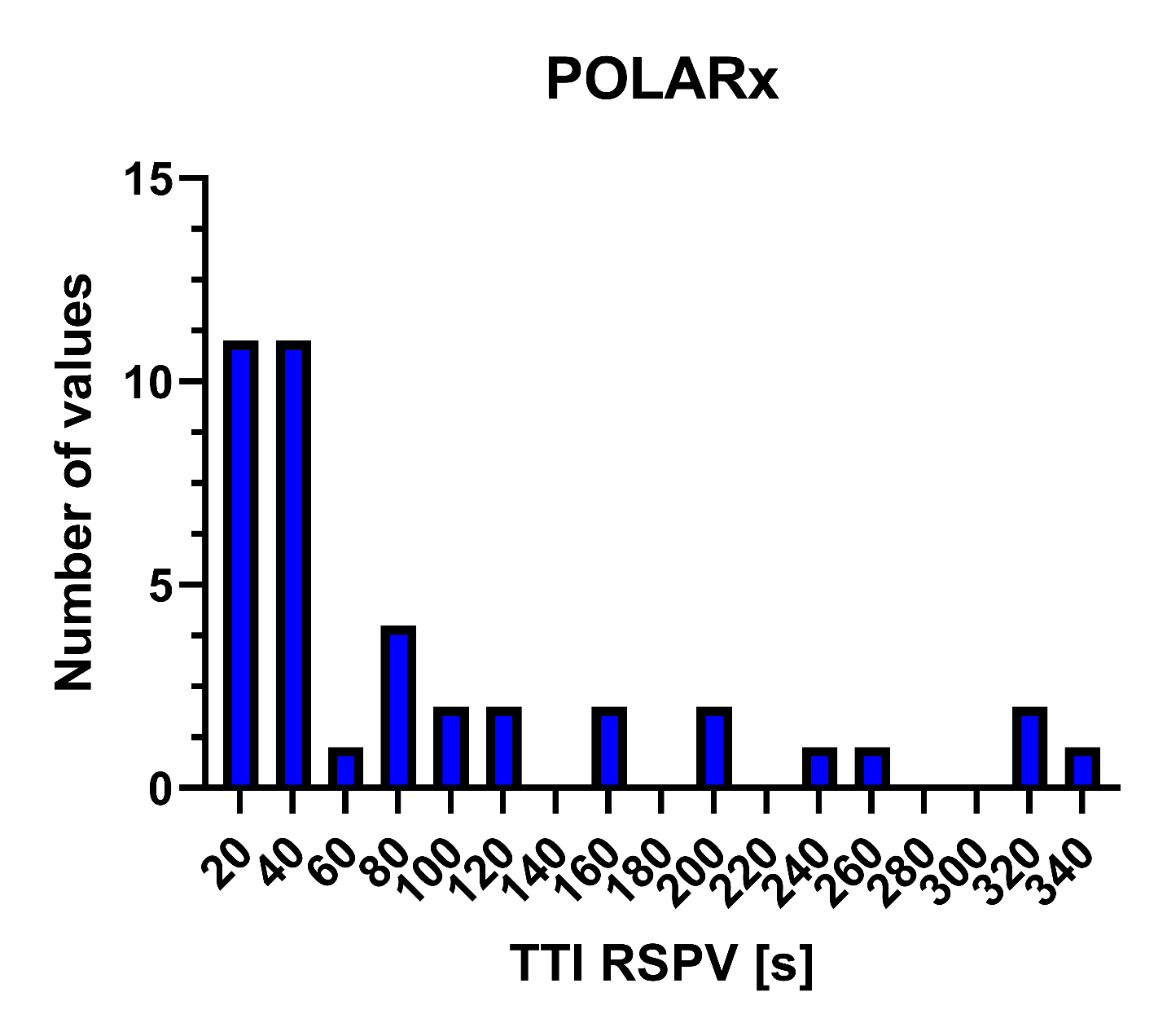

Supplement: Supplementary file 1 — Table S1. Table S2. Table S3. [file JOA3-39-341-s001.docx]
